# Homocysteine Disrupts Balance between MMP-9 and Its Tissue Inhibitor in Diabetic Retinopathy: The Role of DNA Methylation

**DOI:** 10.3390/ijms21051771

**Published:** 2020-03-05

**Authors:** Ghulam Mohammad, Renu A. Kowluru

**Affiliations:** Department of Ophthalmology, Visual and Anatomical Sciences, Wayne State University, Detroit, MI 48202, USA; ed5563@wayne.edu

**Keywords:** diabetic retinopathy, DNA methylation, Epigenetics, Homocysteine, MMPs

## Abstract

High homocysteine is routinely observed in diabetic patients, and this non-protein amino acid is considered as an independent risk factor for diabetic retinopathy. Homocysteine biosynthesis from methionine forms S-adenosyl methionine (SAM), which is a major methyl donor critical in DNA methylation. Hyperhomocysteinemia is implicated in increased oxidative stress and activation of MMP-9, and in diabetic retinopathy, the activation of MMP-9 facilitates capillary cell apoptosis. Our aim was to investigate the mechanism by which homocysteine activates MMP-9 in diabetic retinopathy. Human retinal endothelial cells, incubated with/without 100 μM homocysteine, were analyzed for MMP-9 and its tissue inhibitor Timp1 expressions and interactions, and ROS levels. *Timp1* and *MMP-9* promoters were analyzed for methylated and hydroxymethylated cytosine levels (5mC and 5hmC respectively) by the DNA capture method, and DNA- methylating (Dnmt1) and hydroxymethylating enzymes (Tet2) binding by chromatin immunoprecipitation. The results were confirmed in retinal microvessels from diabetic rats receiving homocysteine. Homocysteine supplementation exacerbated hyperglycaemia-induced MMP-9 and ROS levels and decreased Timp1 and its interactions with MMP-9. Homocysteine also aggravated Dnmts and Tets activation, increased 5mC at *Timp1* promoter and 5hmC at *MMP-9* promoter, and suppressed *Timp1* transcription and activated *MMP-9* transcription. Similar results were obtained from retinal microvessels from diabetic rats receiving homocysteine. Thus, hyperhomocysteinemia in diabetes activates MMP-9 functionally by reducing Timp1-MMP-9 interactions and transcriptionally by altering DNA methylation-hydroxymethylation of its promoter. The regulation of homocysteine could prevent/slow down the development of retinopathy and prevent their vision loss in diabetic patients.

## 1. Introduction

Retinopathy is one of the major microvascular complications of diabetes. Many biochemical, molecular, functional and structural abnormalities are implicated in its development, making it difficult to pinpoint the exact mechanism of its pathogenesis [1]. Cytosolic and mitochondrial free radicals are elevated in the retina and its vasculature, and matrix metalloproteinase-9 (MMP-9) is activated [2,3]. MMP-9 activation is an early event, followed by their translocation inside the mitochondria and damage of the mitochondrial membranes [4]. MMP-9 activity is regulated primarily by its tissue inhibitor, Timp1, and Timp1 binding with MMP-9 catalytic domain blocks access of substrates to the active site of MMP-9. A fine balance between MMP-9 and Timp1 is important in regulating both MMP-9 proenzyme and MMP-9 activation [5]. In diabetes, while retinal MMP-9 expression is upregulated, that of Timp1 is downregulated [6,7].

Diabetic patients routinely have increased levels of a non-protein amino acid, homocysteine [8], and high plasma homocysteine is correlated with decreased retinal nerve layer thickness and the severity of diabetic retinopathy [9,10,11,12]. Our recent study has shown three-fold higher homocysteine levels in the retina from human donors with established diabetic retinopathy compared to retina from nondiabetic human donors [13]. High circulating levels of homocysteine are implicated in many cellular abnormalities, including oxidative stress, mitochondrial fission and the activation of MMP-9 [14,15,16]. The way in which elevated homocysteine regulates retinal MMP-9 activation in diabetic retinopathy is unclear.

Homocysteine is biosynthesized from methionine by S-adenosyl-methionine synthetase, forming S-adenosyl methionine (SAM) [17], and diabetic donors with established retinopathy have impaired machinery to maintain homocysteine levels in the retina and the enzymes critical in transsulfuration and in the re-methylation of homocysteine are reduced [13]. SAM is a critical component of the DNA methylation process [18]. In diabetes, retinal DNA methylating (DNA methyltransferases, Dnmts) and hydroxymethylating enzymes (ten eleven translocases, Tets) are activated and the *MMP-9* promoter undergoes dynamic DNA methylation-hydroxymethylation [19]. The role of homocysteine in the transcriptional regulation of *MMP-9* and *Timp1* remains to be elucidated.

The aim of this study was to investigate the mechanism by which homocysteine activates MMP-9 in diabetic retinopathy. Using retinal endothelial cells, we investigated the effect of homocysteine on MMP-9-Timp1 interactions and on the DNA methylation status of the *MMP-9* and *Timp1* promoters. The results were confirmed in the retinal microvessels from streptozotocin-induced diabetic rats.

## 2. Results

### 2.1. In Vitro: Retinal Endothelial Cells in Culture

**Figure 1:**
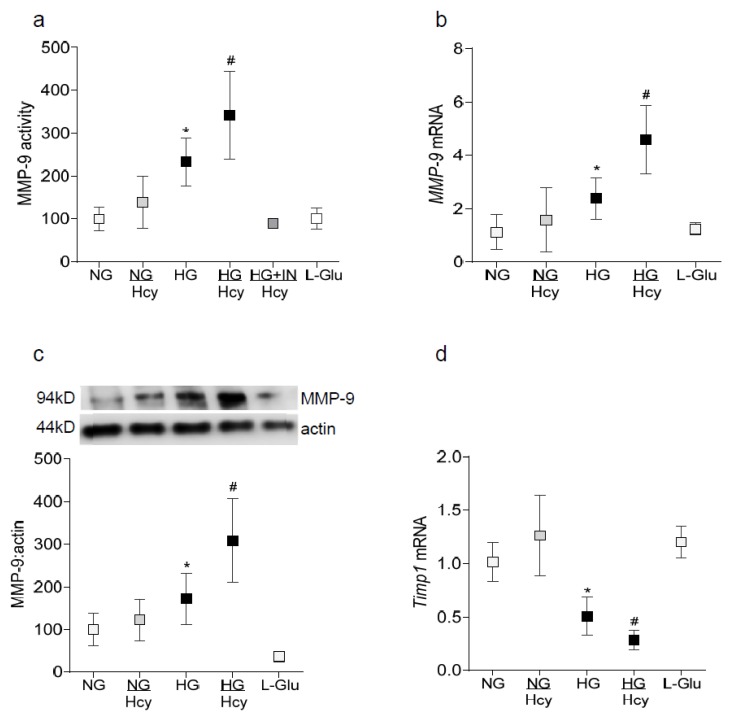
Effect of homocysteine on MMP-9 and Timp1 in retinal endothelial cells. HRECs incubated in the presence or absence of homocysteine in 5 mM or 20 mM glucose were analyzed for MMP-9 (**a**) activity in 30–40 μg protein using an ELISA kit; (**b**) mRNA by q-RT PCR using β-actin as a housekeeping gene, and (**c**) protein expression by the Western blot technique using β-actin as a loading protein. (**d**) *Timp1* mRNA was quantified by q-RT PCR. Each measurement was made in duplicate in three to four cell preparations. NG and HG = HRECs in 5 mM and 20 mM D-Glucose respectively; NG/Hcy and HG/Hcy = cells in 5 mM or 20 mM glucose, in the presence of homocysteine, HG+IN/Hcy = MMP-9 inhibitor pretreated cells in 20 mM D-glucose medium containing homocysteine and L-Glu = 20 mM L-glucose. The values obtained from cells in NG are considered as 100% (activity and protein expression) or one (mRNA), and are represented as mean ± SD. * and ^#^ *p* < 0.05 compared to NG or HG, respectively.

Diabetes activates MMP-9 in the retina and its vasculature [6] and MMP-9 is also activated by homocysteine [14,15]. To investigate the role of homocysteine in the regulation of MMP-9 in diabetic retinopathy, human retinal endothelial cells (HRECs) incubated in the presence of homocysteine were analyzed. As expected, MMP-9 activity was elevated by over 2 fold in the cells exposed to 20 mM D-glucose (HG group), and supplementation of 20 mM D-glucose medium with 100 μM homocysteine (HG/Hcy group) further exacerbated MMP-9 activation (Figure 1a). Although compared to cells in 5 mM D-glucose alone (NG group), MMP-9 activity was increased in cells incubated in 5 mM D-glucose medium supplemented with 100 μM homocysteine (NG/Hcy group) by ~40%, the values failed to achieve any statistical significance. The addition of an MMP-9 inhibitor attenuated high glucose-homocysteine induced exacerbated increase in MMP-9 activity and the values obtained in the HG + IN/Hcy group were not different from those obtained in the NG group. Consistently with the increase in the enzyme activity, MMP-9 gene transcripts and protein expression were also further increased in HG/Hcy group as compared to the HG group (Figure 1b,c). Since Timp1 is an endogenous tissue inhibitor of MMP-9 and regulates its activation, the effect of homocysteine on a glucose-induced decrease in Timp1 expression was examined. As shown in Figure 1d, the addition of homocysteine further decreased *Timp1* mRNA; the values obtained from HG/Hcy group were significantly different from those obtained from the HG group. The incubation of cells in L-glucose, instead of 20 mM D-glucose, had no effect on MMP-9 or Timp1.

**Figure 2:**
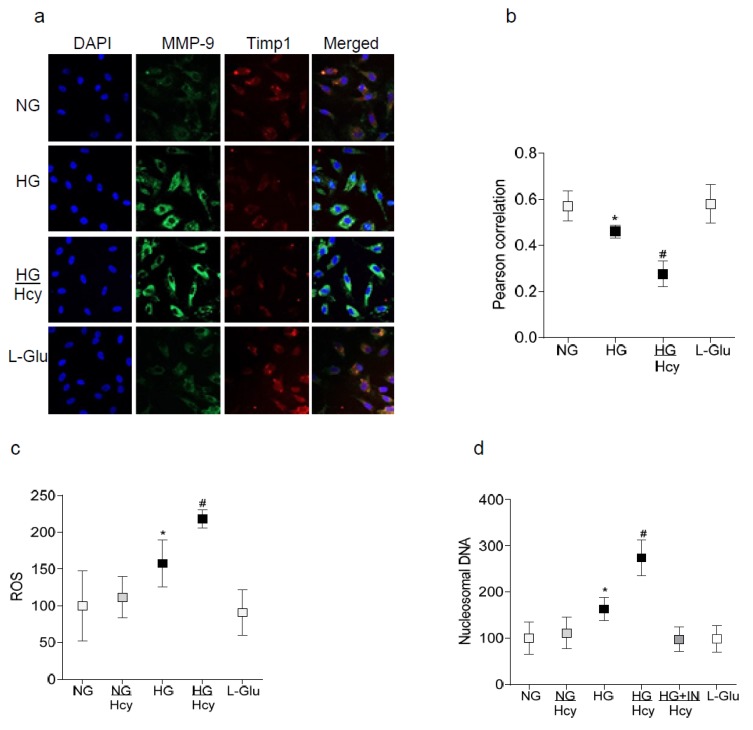
High homocysteine and Timp1-MMP-9 interactions and ROS levels and cell apoptosis. (**a**) Co-localization of Timp1 and MMP-9 was determined by the immunofluorescence technique using Texas Red (red) and Alexa-Flour 488 (green) conjugated secondary antibodies for Timp1 and MMP-9, respectively. After capturing the images in ZEISS at 20× objective magnification with Apotome module, (**b**) the images were calibrated with ZEISS pro-inbuilt software package and modules. Using random region of interest in the outer nuclear area, Pearson’s correlation coefficient was determined. (**c**) ROS levels were quantified in 5–10 μg protein by the DCFDA method, and (**d**) cell apoptosis by using a Cell Death Detection ELISAPLUS kit, the and final product was measured in an ELISA reader at a 405 nm wavelength. Each measurement was made in duplicate or triplicate in four to five different cell preparations. NG and HG = 5 mM and 20 mM glucose respectively, NG/Hcy and HG/Hcy = cells treated with homocysteine in 5 mM or 20 mM glucose, HG+IN/Hcy = MMP-9 inhibitor pretreated cells in 20 mM D-glucose medium containing homocysteine and L-Glu = 20 mM L-glucose. * *p* < 0.05 vs. NG and ^#^ *p* < 0.05 vs. HG.

Timp1 interactions with MMP-9 impede MMP-9 activation [5]; the effect of homocysteine on Timp1-MMP-9 interactions was determined by the immunofluorescence technique. As shown in Figure 2a, compared to cells in normal glucose, the co-localization of Timp1-MMP-9 was decreased by high glucose and this was further reduced when homocysteine was supplemented in a high glucose medium. Pearson’s correlation coefficient was ~50% lower in the HG/Hcy group compared to the HG group (Figure 2b). The incubation of cells in 20 mM L-glucose had no effect on Tipm1-MMP-9 interactions. MMP-9 is a redox-sensitive enzyme [20], and homocysteine increases oxidative stress [14,15,16]; to investigate the role of homocysteine-mediated increase in reactive oxygen species (ROS) in exacerbated MMP-9 activation, ROS levels were quantified. As shown in Figure 2c, high glucose-induced increase in ROS was further intensified by the presence of homocysteine in the medium and the values obtained in HG and HG/Hcy groups were significantly different from each other. Furthermore, the activation of MMP-9 in hyperglycemic milieu activates the apoptosis machinery, increasing capillary cell apoptosis [6,21]. Consistently with the activation of MMP-9, homocysteine supplementation further increased glucose-induced apoptosis of retinal capillary. To confirm that the exacerbation of high glucose-induced retinal capillary cell apoptosis by homocysteine is via MMP-9 activation, cells in the HG+IN/Hcy group were analyzed; The MMP-9 inhibitor prevented the high glucose+ homocysteine-mediated aggravated apoptosis of retinal capillary cells. The values obtained from the cells in the NG and HG+IN/Hcy groups were not different from each other (Figure 2d).

**Figure 3:**
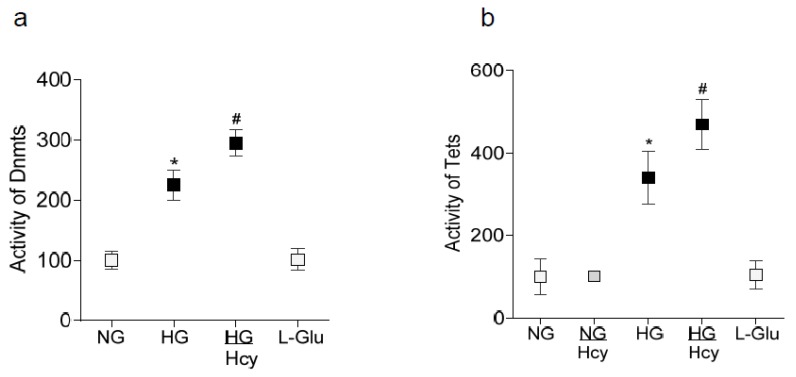
Effect of homocysteine on the activities of Dnmts and Tets. HRECs incubated in the presence or absence of homocysteine were analyzed for the activities of (**a**) Dnmts in 5 μg nuclear protein using a DNA Methyltransferase Activity/Inhibition assay kit, and (**b**) Tets in 10–15 μg nuclear protein using a TET Activity/Inhibition Assay Kit. The values obtained from cells in NG are considered 100%. Values are represented as the mean ± SD from four to five cell preparations, with each measurement made in duplicate. * and ^#^ *p* < 0.05 compared to NG and HG respectively.

Homocysteine plays a crucial role in DNA methylation, and in diabetes, the machinery responsible for maintaining DNA methylation status is upregulated and the *MMP-9* promoter is hydroxymethylated [19]. To investigate the mechanism(s) responsible for transcriptional activation and repression of *MMP-9* and *Timp1*, respectively, the effect of homocysteine on the activation of Dnmts and Tets was determined. Compared to cells in normal glucose, the activities of both Dnmts and Tets were increased significantly by high glucose and the increase in activities was further intensified by the addition of homocysteine; HG and HH/Hcy group values were significantly different from each other (Figure 3a,b).

**Figure 4:**
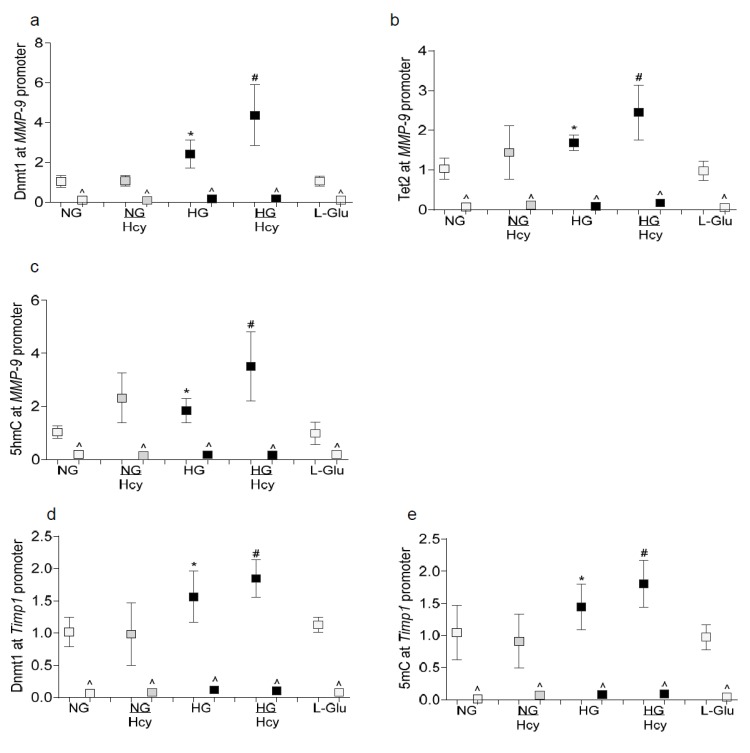
Effect of homocysteine on DNA methylation status of *MMP-9* and *Timp-1* promoters. The promoter region of *MMP-9* was analyzed for (**a**) Dnmt1 and (**b**) Tet2 binding by the ChIP technique and (**c**) 5hmC levels by the hydroxymethylated DNA immunoprecipitation method. (**d**) Dnmt1 binding and (**e**) 5mC at *Timp1* promoter were quantified by the ChIP technique and the methylated DNA immunoprecipitation method respectively. IgG (^) was used as an antibody control. Each measurement was made in duplicate in three to four different cell preparations. NG and HG = 5 mM and 20 mM glucose, respectively, NG/Hcy and HG/Hcy = cells treated with homocysteine in 5 mM or 20 mM glucose, L-Glu = 20 mM L-glucose. * *p* < 0.05 vs. NG and ^#^ *p* < 0.05 vs. HG.

Consistently with increase in the activities of Dnmts and Tets, the binding of both Dnmt1 and Tet2 (the only isoforms activated in the retina in diabetes [19], at the *MMP-9* promoter was further exacerbated in the HG/Hcy group, and 5-hydroxymethyl cytosine (5hmC) levels were significantly higher compared to those in the HG group (Figure 4a–c). Compared with the cells exposed to normal glucose, high glucose also increased Dnmt1 binding and 5-methyl cytosine (5mC) levels at the *Timp1* promoter by over 50% and the addition of homocysteine further exacerbated these glucose-induced increases; the values in the HG and HG/Hcy groups were significantly different from each other (Figure 4d,e). The incubation of cells in 20 mM L-glucose alone had no effect on the DNA methylation status of either *MMP-9* or *Timp1*. The values obtained from the IgG antibody control (^) were >0.5% of those values obtained from the MMP-9 or Timp1 antibodies.

### 2.2. In Vivo Model: Rat Retinal Microvasculature

**Figure 5:**
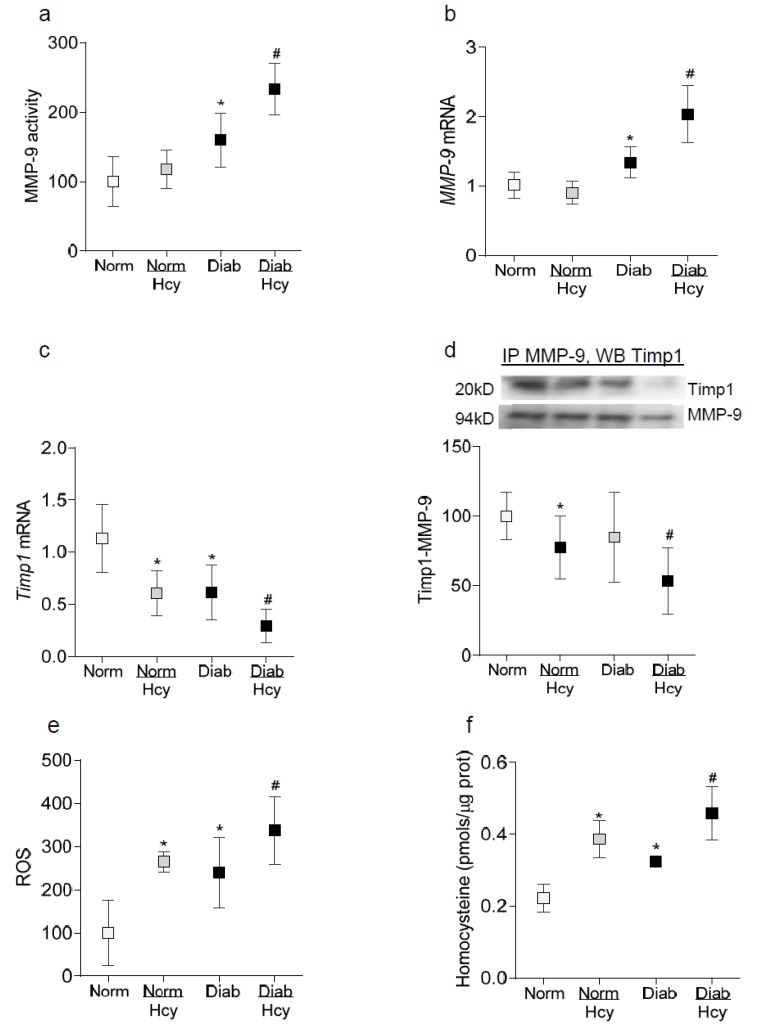
Effect of homocysteine on diabetes-induced alterations in MMP-9, Timp1, Timp-MMP-9 interactions and ROS levels. Retinal microvessels from diabetic rats, with or without homocysteine supplementation were analyzed for (**a**) MMP-9 activity spectrophotometrically, (**b**,**c**) *MMP-9* and *Timp1* gene transcripts by q-RT PCR using β-actin as a housekeeping gene, (**d**) Timp1-MMP-9 interactions by co-immunoprecipitation technique by first immunoprecipitating MMP-9, followed by Western blotting for Timp1, and (**e**) ROS levels fluorometrically using DCFDA. (**f**) Homocysteine levels were quantified in retinal homogenate spectrophotometrically. Each measurement was made in duplicate in 5–7 rats/group, and the values are represented as mean ± SD. Norm and Diab = normal control and diabetes respectively, and Norm/Hcy and Diab/Hcy = normal or diabetic rats receiving homocysteine administration. * *p* < 0.05 vs. normal and ^#^ *p* < 0.05 vs. diabetes.

To confirm our in vitro results in an in vivo model, retinal microvessels from diabetic rats receiving homocysteine were analyzed. Consistently with the results from isolated cells, the administration of homocysteine to rats significantly exacerbated diabetes-induced increase in MMP-9 activity and its gene transcripts and further decreased *Timp1* transcripts and ROS levels. Although the administration of homocysteine to the nondiabetic control rats significantly decreased Timp1 expression and ROS levels, it had no significant effect on MMP-9 expression. Compared to normal rats receiving homocysteine or diabetic rats without homocysteine, in the same group of diabetic rats receiving homocysteine, interactions between Timp1 and MMP-9 were also further decreased (Figure 5a–e). Figure 5f is included to show a significant increase in homocysteine levels in the retina of rats receiving supplemental homocysteine.

**Figure 6:**
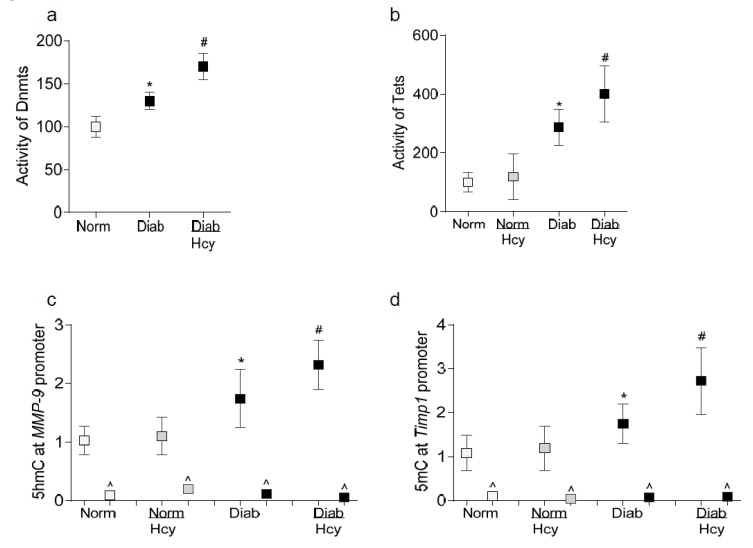
Homocysteine and DNA methylation status of *MMP-9* and *Timp1* promoters. Activities of (**a**) Dnmts and (**b**) Tets was quantified in retinal nuclear fraction using *DNA Methyltransferase or Hydroxymethyltransferase* Activity/Inhibition kits. The values from normal control rats were considered as 100%. The levels of (**c**) 5hmC at the MMP-9 promoter, and (**d**) 5mC at the Timp1 promoter were measured using by hydroxymethylated and methylated DNA immunoprecipitation methods, respectively. As an antibody control, IgG (^) was used. The vales from normal control rats were considered as one. Each measurement was made in duplicate in 5–6 rats/group. * *p* < 0.05 compared to normal and ^#^ *p* < 0.05 compared to diabetes.

In accordance with the results from the in vitro model, homocysteine supplementation further activated both Dnmts and Tets; the values obtained from diabetic rats receiving homocysteine were significantly higher than the rats receiving no homocysteine (Figure 6a,b). Similarly, 5hmC and 5mC levels at *MMP-9* and *Timp1* promoters, respectively, were further elevated in retinal microvessels from diabetic rats receiving homocysteine compared to diabetic rat without homocysteine (Figure 6c,d). IgG antibody control (^) values were >0.5% of the values from *MMP-9* or *Timp1* antibodies. Normal rats with or without homocysteine had similar 5hmC levels at *MMP-9* and 5mC at *Timp1* promoters.

## 3. Discussion

In the development of diabetic retinopathy, although hyperglycemia is the major instigator, homocysteine dysmetabolism is also considered as an important factor. Clinical studies have documented that elevated homocysteine levels represent an independent risk factor for diabetic retinopathy and a strong association between elevated homocysteine levels and retinal endothelial dysfunction, blood-retinal barrier break down and ganglion cell death was observed in diabetes [8,9,10,11,12]. Here, our exciting results from both in vitro and in vivo models show that although in hyperglycemic milieu, MMP-9 is activated in retinal microvasculature and its endogenous inhibitor Timp1 is downregulated, the addition of homocysteine further increases ROS levels and decreases interactions between Timp1 and MMP-9, exacerbating the MMP-9 activation and apoptosis of capillary cells. Homocysteine supplementation in a hyperglycemic milieu also aggravates the activation of the DNA methylation machinery, which further downregulates the transcription of *Timp1* and upregulates that of *MMP-9*. This results in a decreased availability of Timp1 to interact with the excess MMP-9 and facilitates MMP-9 activation. Thus, elevated levels of homocysteine in diabetes, by altering ROS and Timp1 levels and activating DNA methylating- hydroxymethylating enzymes, regulate both the functional and transcriptional activation of MMP-9, an enzyme which is intimately associated with mitochondrial dysfunction-capillary cell apoptosis in diabetic retinopathy.

Homocysteine has a -SH group like thiols (RSH) and this group is susceptible to oxidation in the presence of metal catalysts and molecular oxygen [22]. Moreover, homocysteine can also form Hcy-thiolactone, exacerbating oxidative stress, and it can produce hydrogen peroxide [23,24]. A high homocysteine production reduces hydrogen sulfide (H_2_S), and imbalance between homocysteine and H_2_S increases oxidative stress [25,26]. MMPs are produced in inactive zymogen form and the cleavage of the pro-peptide region forms biologically active MMPs. MMP-9 activity is further regulated by the inhibition of the active forms by Timp1, which allows the release of un-complexed catalytically active component of MMP-9. MMP-9 has crucial cysteine residues within its DNA-binding domain and the enzyme itself is sensitive to oxidative stress [27,28]; our previous studies have shown that retinal MMP-9 activation in diabetes can be ameliorated by the regulation of oxidative stress [20,29]. Timp1 also has two domains, with each domain stabilized by three disulfide bonds, and its N-domain is a stable native domain with inhibitory activity against MMPs [30]. Here, our results show that homocysteine supplementation adds to the diabetes-induced increase in ROS generation, increasing MMP-9 activity and decreasing Timp1 levels and also its interactions with MMP-9. In support, others have shown that high glucose and homocysteine synergistically affect the metalloproteinases and their tissue inhibitors in human fibroblasts [31]. The activation of MMP-9 in diabetes damages retinal mitochondria, releasing cytochrome c into the cytosol and accelerating apoptosis of retinal capillary cells [6,32]. Our results clearly show that homocysteine also exacerbates capillary cell apoptosis, that the cells already experience in hyperglycemic conditions.

Homocysteine production from methionine yields SAM, which is a major methyl donor for DNA methylation, a process mediated by Dnmts [33]. Homocysteine is shown to be associated with global DNA methylation in glomerular and tubulointerstitial fibrosis, and cystathionine-β-synthase deficient mice have an increased protein expression of Dnmts [34]. Furthermore, homocysteine increases Dnmt activity in endothelial cells [35] and pretreatment with sodium hydrosulfide attenuates homocysteine-induced increased Dnmt1 and Dnmt3a [36]. In diabetes, the enzymes responsible for both DNA methylation and hydroxymethylation, Dnmts and Tets, are activated, and DNA methylation is implicated in the transcriptional regulation of many key genes associated with diabetic retinopathy [32,37]. Human *Timp1* promoter has a 1843-1968bp CpG island region, and abnormal *Timp1* DNA methylation is seen in glomerular interstitial fibrosis [34]. Furthermore, our previous work has shown that in diabetes, *MMP-9* transcription is regulated by dynamic DNA methylation; although the activation of Dnmt increases its binding, concurrent activation of the hydroxymethylation machinery keeps the *MMP-9* promoter hypomethylated [19,38,39]. Here, we demonstrate that homocysteine further increases hyperglycemia-mediated activation of both Dnmts and Tets. This facilitates the binding of Dnmt1 and Tet2 to the *MMP-9* promoter, resulting in a net increase of hydroxymethylated cytosine and the transcriptional activation of *MMP-9*. Consistently with the altered DNA methylation status of *MMP-9* promoter in hyperglycemic-high homocysteine medium, Dnmt1 binding at the *Timp1* promoter is also further increased and methylated cytosine levels are elevated, exacerbating *Timp1* transcriptional suppression.

The results presented here show that although homocysteine levels are also significantly elevated in the retina of normal rats receiving supplemental homocysteine and these levels are not different from those obtained from diabetic rats without additional homocysteine supplementation, the functional and transcriptional regulation of MMP-9 in normal rats and normal rats with homocysteine are not different from each other. Furthermore, the values obtained in these two normal rat groups were significantly different than those obtained from diabetic and diabetic +homocysteine groups. These results clearly suggest that hyperglycemic milieu could serve as one of the exacerbating factors in intensifying the detrimental effects of high-circulating homocysteine.

We recognize that the present study is focused on MMP-9, but homocysteine also activates MMP-2, another member of the gelatinase family of MMPs [40,41]. Consistently with MMP-9, the activation of MMP-2 is also an early event in the pathogenesis of diabetic retinopathy, which precedes mitochondrial damage [4,42], and we cannot rule out the role of homocysteine in functional and transcriptional regulation of MMP-2 in diabetic retinopathy, but this is beyond the scope of the present study.

In conclusion, our in vitro and in vivo models clearly show that the presence of homocysteinemia in a diabetic environment, by increasing ROS levels and decreasing Timp1-MMP-9 interactions, functionally activates MMP-9, and by further facilitating alterations in the DNA methylation-hydroxymethylation process, increases *MMP-9* transcription and represses *Timp1* transcription. A decrease in Timp1 fails to properly interact with increased levels of MMP-9 and aggravates MMP-9 activation, producing a synergistic effect for an already compromised retinal vasculature in diabetes. Thus, the regulation of homocysteine levels by diabetic patients could prevent/slow down the development of retinopathy and prevent their vision loss.

## 4. Materials and Methods

Retinal endothelial cells: Primary HRECs were purchased from Cell Systems Corporation (Cat. No. ACBRI 181, Cell Systems Corp, Kirkland, WA, USA), and cultured in Dulbecco’s modified Eagle medium (DMEM)-F12 supplemented with 15 μg/mL endothelial cell growth supplement, 10% heat-inactivated fetal bovine serum, and 1% each insulin- transferrin- selenium-Glutamax and antibiotic/antimycotic. Cells were grown in a humidified environment of 95% O_2_ and 5% CO_2_ at 37 °C, and confluent cells from the 6th to the 9th passage were incubated in 20 mM D-glucose for 96 hours in the absence or presence L-homocysteine thiolactone hydrochloride (Hcy,100 μM; Cat. No. S784036; Sigma-Aldrich, St. Louis, MO, USA) [43]. The choice of using 100 μM homocysteine was based on our initial experiments; we had more consistent results with 100 μM homocysteine than 50 μM homocysteine. A group of cells were pretreated with 4 nM of MMP-9 inhibitor (MMP-9 inhibitor-I, CAS 1177749-58-4, Sigma-Aldrich) for eight hours before being incubated in high glucose medium supplemented with homocysteine (HG+IN/Hcy group) for 96 h [44]. Cells incubated in 5 mM D-glucose in the absence or presence of Hcy (NG and NG/Hcy groups) served as controls. For osmotic/metabolic control, parallel incubations were conducted using 20 mM L-glucose (L-Glu), instead of 20 mM D- glucose [45].

### 4.1. Diabetic Rats

Wistar rats (male, 200 g) were made diabetic by streptozotocin (55 mg/kg BW, ip) [46]. Soon after induction of diabetes, the rats were divided into two groups, rats in group 1 received intraperitoneal injection of DL-homocysteine (Hcy, Cat. No. 44925; Sigma-Aldrich), dissolved in PBS, at a dose of 100 mg/kg BW, every other day (Diab/Hcy). Rats in group 2 remained diabetic, without any supplementation (Diab). Eight weeks after initiation of the experiments, the rats were sacrificed and their retina was collected. Age-matched normal rats without or with homocysteine administration (Norm and Norm/Hcy groups respectively) served as controls. The treatment of animals conformed to the ARVO’s Statement for the Use of Animals in Ophthalmic and Vision Research, and by the Wayne State University’s Institutional Animal Care and Use Committee (protocol # IACUC-18-02-0525; approval date, 30 May 2018).

### 4.2. Retinal Microvessels

Freshly isolated retina was incubated in ~10 mL distilled water for 1 h at 37 °C in a shaker water bath. The nonvascular tissue was then gently removed under a dissecting microscope using Pasteur pipette, and the microvessel preparation was used for analysis. As reported previously, the microvessel preparations had a normal complement of nuclei and were generally free of nonvascular contaminants [45,47].

### 4.3. Homocysteine Levels

Retinal homogenate (10–15 µg protein) was utilized to quantify homocysteine levels spectrophotometrically, using an ELISA kit from Cell Bio Labs Inc. (Cat No. STA-670, San Diego, CA, USA), as described previously [13].

### 4.4. Gene Transcripts

Total RNA was extracted from HRECs or retinal microvessels by TRIZOL reagent (Invitrogen, Carlsbad, CA), and quantitative real-time PCR (q-RT PCR) was performed using SYBR green master mix (Applied Biosystems) and gene- and species-specific primers (Table 1). The specific products were confirmed by SYBR a green single melt curve analysis with the ABI750 Real Time PCR System. *β-actin* was used as the housekeeping gene, and relative fold change was calculated using the delta-delta Ct method [45].

### 4.5. Western Blotting

Protein samples (30 μg) were separated on a 4–20% gradient acrylamide gel (Bio-Rad, Hercules, CA), and then transferred onto a nitrocellulose membrane. After blocking at room temperature in 5% non-fat milk for 1 h, the membranes were incubated overnight with MMP-9 antibody (Cat No. Ab38898, Abcam, 1:1000 dilution) at 4 °C. The membranes were then incubated with HRP-conjugated secondary antibody at room temperature for 1 h. Protein bands were visualized by enhanced chemiluminescence (Thermo Fisher Scientific Inc., Rockford, IL, USA). β-Actin (Cat No. A-5316, Sigma-Aldrich, 1:3000 dilution) was used as a loading protein.

### 4.6. Activities of Enzymes

The activity of MMP-9 was determined in 30–40 μg protein using an ELISA kit (Cat No. RPN2634, Amersham, Buckinghamshire, UK) and the final product was quantified spectrophotometrically at 405 nm using chromogenic peptide substrate [48].

The enzyme activities of Dnmts and Tets were quantified in the nuclear fraction (5–15 μg protein) using E*piQuik™ DNA Methyltransferase Activity/Inhibition and* Epigenase 5mC-Hydroxylase TET Activity/Inhibition Assay Kits, respectively (Cat. Nos. P-3001 and P-3087; EPIGENTEK, Farmingdale, NY, USA), as described previously [19].

### 4.7. Immunofluorescence

The cellular localization of MMP-9 and Timp1 was performed by the immunofluorescence technique using primary antibodies against MMP-9 (ab38898; Abcam, 1:200 dilution), and Timp1 (Cat. No. 1M41; Calbiochem, Nottingham, UK, 1:200 dilution), and DyLight green- labeled for MMP-9 or Texas red-labeled for Timp1 conjugated secondary antibodies. DAPI(4′,6-diamidino-2-phenylindole) containing mounting media (Vector Laboratories, Burlingame, CA) was employed to visualize the nucleus and the slides were examined using the Zeiss ApoTome fluorescence microscope at 20× magnification (Carles Zeiss, Inc., Chicago, IL, USA).

### 4.8. Co-Immunoprecipitation

Co-immunoprecipitation of MMP-9 and Timp1 was performed in 100 μg retinal homogenate by first immunoprecipitating MMP-9, followed by incubating with Protein A/G Plus agarose beads (suspended in the lysis buffer). After washing the beads, the proteins were separated on an SDS-PAGE and were immunoblotted with anti-Timp1 antibody (Cat. No. 1M41; Calbiochem, 1:500 dilution).

### 4.9. Reactive Oxygen Species

Total ROS levels were quantified fluorometrically using 2′,7′-dichlorofluorescein diacetate (DCFDA; Sigma-Aldrich). Briefly, 5 μg proteins was incubated with 4 μM DCFDA for 10 min and the fluorescence was measured at 485 nm and 530 nm as excitation and emission wavelengths, respectively [49].

### 4.10. Apoptosis of Retinal Endothelial Cells

Cell apoptosis was measured using the Cell Death Detection ELISAPLUS kit from Roche Diagnostics (Cat. No. 11774425001, Indianapolis, IN) according to the manufacturer’s instructions. Measurements were made using an ELISA reader at 405 nm wavelength and the results were calculated as fold change considering the values obtained from cells in normal glucose as one [46].

### 4.11. Methylated/Hydroxymethylated Cytosine

The levels of 5mC and 5hmC were quantified in the total genomic DNA by MethyLamp Methylated and Hydroxymethylated DNA capture Kits (Cat. No. P-1015 and Cat. No. P-1038 respectively, EPIGENTEK), as reported previously [45,46], using the primers targeting *MMP-9* and *Timp1* promoter regions (Table 1).

### 4.12. Chromatin Immunoprecipitation (ChIP)

ChIP was performed in the cross-linked HRECs or microvessels, sonicated in ChIP lysis buffer. The Protein–DNA complex (100 μg) was immunoprecipitated with antibodies against either Dnmt1 or Tet2 (Cat. Nos. Ab13537 and Ab135087 respectively, Abcam, Cambridge, MA, USA). Normal rabbit IgG (Cat. No. Ab171870, Abcam) was used as an antibody control. Protein A/G agarose beads (Cat No. sc-2003, Santa Cruz Biotechnology, Santa Cruz, CA) were employed to pull down the antibody–chromatin complexes. Protein-associated DNA was recovered by incubating the eluent at 65 °C, purified by phenol extraction, and precipitated with ethanol. Dnmt1/Tet2 binding at *MMP9* and *Timp1* promoter was quantified by q-PCR using primers specific for their promoters [46].

### 4.13. Statistical Analysis

Data are presented as the mean ± SD. A comparison between groups was made using one-way ANOVA followed by Dunn’s *t*-test; *p* < 0.05 was considered statistically significant.

## Figures and Tables

**Table 1:** Primer sequence.

Gene	Sequence
Human	
*MMP-*9	Fwd- CACTGTCCACCCCTCAGAGC
	Rev- GCCACTTGTCGGCGATAAGG
*Timp1*	Fwd- GAAGAGCCTGAACCACAGGT
	Rev- CGGGGAGGAGATGTAGCAC
*MMP-9* promoter	Fwd- GAGTCAGCACTTGCCTGTCA
	Rev- CTGCTGTTGTGGGGGCTTTA
*Timp1* promoter	Fwd- CTGATGGTGGGTGGATGAGTAATG
	Rev- GCCTGAGCGCTAGAGGATAAATG
*β-Actin*	Fwd- AGCCTCGCCTTTGCCGATCCG
	Rev- TCTCTTGCTCTGGGCCTCGTCG
Rat	
*MMP-*9	Fwd- GAAGCAGCTGTCCCTGCCCC
	Rev- GATGGTGCCACTTGAGGTCG
*Timp1*	Fwd- GTTTCCGGTTCGCCTACACC
	Rev- GGGCTCAGATTATGCCAGGG
*MMP-9* promoter	Fwd- GACTGTGGGCAGGGCATAAA
	Rev- GGGTGAAGCAGAATTTGCGG
*Timp1* promoter	Fwd- GCAGTGGGTGGATGAGTAAT
	Rev- GACTGGCGGTGGAGAATA AA
*β-Actin*	Fwd- TCTGTGTGGATTGGTGGCTCTA
	Rev- AAC AGTCCGCCTAGAAGCATTTG
